# Novel Strategies for the Improvement of Stem Cells' Transplantation in Degenerative Retinal Diseases

**DOI:** 10.1155/2016/1236721

**Published:** 2016-05-18

**Authors:** Simona Delia Nicoară, Sergiu Șușman, Oana Tudoran, Otilia Bărbos, Gabriela Cherecheș, Simion Aștilean, Monica Potara, Olga Sorițău

**Affiliations:** ^1^Department of Ophthalmology, “Iuliu Hațieganu” University of Medicine and Pharmacy, 8 Victor Babeș Street, 400012 Cluj-Napoca, Romania; ^2^Department of Histology, “Iuliu Hațieganu” University of Medicine and Pharmacy, 8 Victor Babeș Street, 400012 Cluj-Napoca, Romania; ^3^“Ion Chiricuță” Institute of Oncology, Laboratory of Cell Biology and Radiobiology, 34-36 Ion Creangă Street, 400010 Cluj-Napoca, Romania; ^4^Nanobiophotonics and Laser Microspectroscopy Center, Interdisciplinary Research Institute on Bio-Nano-Sciences, Babeș-Bolyai University, 42 Treboniu Laurian Street, 400271 Cluj-Napoca, Romania; ^5^Department of Biomolecular Physics, Faculty of Physics, Babeș-Bolyai University, 1 Mihail Kogalniceanu Street, 400084 Cluj-Napoca, Romania

## Abstract

Currently, there is no cure for the permanent vision loss caused by degenerative retinal diseases. One of the novel therapeutic strategies aims at the development of stem cells (SCs) based neuroprotective and regenerative medicine. The main sources of SCs for the treatment of retinal diseases are the embryo, the bone marrow, the region of neuronal genesis, and the eye. The success of transplantation depends on the origin of cells, the route of administration, the local microenvironment, and the proper combinative formula of growth factors. The feasibility of SCs based therapies for degenerative retinal diseases was proved in the preclinical setting. However, their translation into the clinical realm is limited by various factors: the immunogenicity of the cells, the stability of the cell phenotype, the predilection of SCs to form tumors* in situ*, the abnormality of the microenvironment, and the association of a synaptic rewiring. To improve SCs based therapies, nanotechnology offers a smart delivery system for biomolecules, such as growth factors for SCs implantation and differentiation into retinal progenitors. This review explores the main advances in the field of retinal transplantology and applications of nanotechnology in the treatment of retinal diseases, discusses the challenges, and suggests new therapeutic approaches in retinal transplantation.

## 1. Introduction

The retinal photoreceptors and the underlying retinal pigmented epithelium (RPE) form a functional unit. The destabilization of this relationship leads to the loss of photoreceptors and subsequent decrease in vision with the ultimate stage of blindness. Clinically, a variety of retinal diseases fall into this pattern: retinitis pigmentosa (RP), Stargardt disease, and age-related macular degeneration (AMD) [1]. At the present moment, there is no cure for the permanent loss of vision associated with degenerative retinal diseases. The impact is significant on the quality of patients' lives and it involves all the age groups. This situation provides an important impetus in the search for new therapies [2]. The common denominator in the above-mentioned diseases is the loss of the neural cells (photoreceptors, interneurons, and retinal ganglion cells) and of the essential supporting cells (retinal pigmented epithelium). Therefore, the novel therapeutic strategies aim at the development of neuroprotective and regenerative strategies, particularly by addressing cellular therapies with stem cells [3].

There is a place for stem cells based treatments in ischemic retinopathies, too, such as diabetic retinopathy (DR) and retinal vascular occlusions. The principle of cell therapy in ischemic retinopathies is to acquire a vascular endothelial cell phenotype and incorporate it into the resident vessels, aiming to restore the vascularization of the damaged tissue. To date, the best option for vascular cell therapy is the endothelial progenitor cell (EPC), but more research should be focused on this area in the coming years [4]. Stem cells represent a potential and promising therapy in DR, as they can repair the damaged retinal tissue, but they can also differentiate into endothelial cells, with subsequent formation of intraretinal capillaries [5].

Some of the arguments supporting the use of stem cells in retinal regeneration are (1) the low immunogenicity of SC and (2) the privileged immunological status of the eye. All these facts are very promising in theory, but many studies proved poor survival of stem cells transplanted in the subretinal space [6].

By consequence, intense study is required regarding stem cell transplantation, in order to identify the reasons for failure, develop new methods to improve their acceptance by the host organism, keep them functional, and guide their orientation towards the desired type of cells. Nanotechnology can be a valuable tool in increasing the bioavailability of biomolecules and therapeutic agents with beneficial effects in retinal regeneration, due to the ability of nanoparticles to pass through the eye barriers (blood-retinal barrier) [7]. Most studied applications of nanoparticles in relationship with stem cells included cells' tracking, imaging, biosensors, or carriers for bioactive molecules involved in stem cell differentiation [8].

## 2. Types of Cells Used for Retinal Regeneration

Some specific anatomical and immunological aspects of the eye recommend and encourage the use of cellular therapies: easy access for the therapeutic procedure and the immune-privileged status of the vitreous cavity and of the anterior chamber [9]. There are three main types of cells that can be used for retinal regeneration: embryonic stem cells (ESC), adult stem cells (MSC), and induced pluripotent stem cells (iPSC) [10]. Their advantages and disadvantages are presented in Table 1.

Stem/progenitor cells can be isolated from various tissues with the aim to differentiate them into retina-specific cells. The main sources are the embryo (ESC), the bone marrow (BM), the region of neuronal genesis, and the eye (the ciliary body epithelium and marginal zone, the iris, and the retina) [11].

The immune privilege of the subretinal space is not absolute. Furthermore, the RPE cells express HLA class II antigens. Therefore, the immune suppression is required in order to reduce the likelihood of the immune rejection of the transplanted allogeneic hESC-derived RPE [3].

## 3. Factors Influencing the Differentiation Process of Stem Cells

The origin of stem cells may determine the direction in which they will differentiate, according to a certain configuration of growth factors or chemical modulators. For instance, stem cells isolated from the ciliary body differentiated into retinal neurons, in the presence of insulin and Epidermal Growth Factor (EGF), but also into bipolar cells, retinal ganglion cells, and photoreceptors, in the presence of bFGF (basic Fibroblast Growth Factor) and GDNF (Glial-Derived Growth Factor). Isolated cells from Müller glia can differentiate into bipolar, rod photoreceptor cells, and amacrine cells in the presence of BDGF (Brain Derived Growth Factor), EGF, retinoic acid (RA), and activin A [7].

Embryonic stem cells/induced pluripotent stem cells (ESCs/iPSCs) and endogenous retinal stem cells such as Müller glial cells, ciliary pigment epithelial cells, and retinal pigment epithelial derived cells can differentiate into multiple types of retinal cells and can replace lost photoreceptors and RPE cells [12, 13]. Mead et al. (2015) concluded in their review that ESCs/iPSCs are more indicated to replace lost retinal cells, whereas MSCs are more useful as a source of neuroprotective paracrine factors for the optic nerve, in degenerative eye diseases [13].

## 4. Key Elements for a Successful Transplantation of Stem Cells

In cell therapies, there are some important steps for a successful transplantation: (1) origin of cells (embryonic stem cells, adult MSCs from different sources, iPSC); (2) route of administration, as less invasive as possible; (3) local microenvironment, represented by (a) extracellular matrix signals and (b) a proper combinative formula of growth factors.

*(1) Choosing the Cells*. Several studies have investigated various stem cell types as potential sources for retinal transplantation, including ESCs, adult stem/progenitor cells, and, more recently, iPSCs [14].

*(a) The Progenitor Cells of the Retina (Neural Precursor Cells)*. They do have the potential to constitutively replace the different cells of retinal-like neurons, photoreceptors, and glial cells [15, 16]. This approach could be useful in developing and studying the pathobiology of stem cells in health and disease. The main issue is represented by their limited availability, because of the difficulties in obtaining them. The ciliary body and iris pigment progenitor cells contain a mitotically quiescent population of neural progenitors that proliferate to make neural stem cells, with a potential for self-renewal [17, 18]. Experiments have documented the incorporation of these cells into injured retina, but not into the normal one, suggesting that functional integration is possible only in damaged tissues.

*(b) Mesenchymal Stem Cells (MSC)*. It was reported that MSCs can be induced* in vitro* into cells expressing photoreceptor lineage-specific markers using activin A, taurine, and Epidermal Growth Factor [19]. In addition, an* in vivo* animal model demonstrated that MSCs injected in the subretinal space can slow down retinal cell degeneration, integrate into the retina, and differentiate into photoreceptors, in RCS rats [20].

*(c) Embryonic Stem Cells*. They were also used in retinal diseases. In 2011, the first phase I/II clinical trial, testing the safety of hESC-derived retinal cells to treat patients with Stargardt's Macular Dystrophy (ClinicalTrials.gov Identifier: NCT01345006) and AMD (ClinicalTrials.gov Identifier: NCT01344993) was initiated [21]. At 4 months, the hESC-derived RPE cells showed no signs of hyperproliferation, tumor genesis, ectopic tissue formation, or apparent rejection and it proved safety evidence for medium-term to long-term follow-up [21]. The future therapeutic goal will be to treat patients earlier in the disease process, increasing the likelihood of photoreceptor and central vision rescue.

The short-term survival of transplanted photoreceptor progenitor cells raises many questions about cell delivery, mode of administration, indications to combine cell therapy with biomaterials, and cell delivery systems [22–24].

The ineffectiveness of cell therapies in retinal diseases has several explanations: (1) RPE cells represent an adherent monolayer of cells and need a compatible matrix following transplantation; (2) the basal lamina layer of Bruch's membrane is damaged in advanced retinal diseases; (3) transplanted cells fail to form a polarized RPE monolayer and this lack of cell-to-cell contact may lead to the transition of RPE cells towards inappropriate phenotypes, such as epithelial-mesenchymal cells [23, 25].

*(2) Mode of Administration*. The route of administration has to be as less invasive as possible. Stem cells homing into the injured eyes is not fully understood. Studies have shown that stem cells' migration and organ-specific homing are regulated by chemokines and their receptors. The expression of CXCR4 has been reported on embryonic stem cells [26] and adult stem cells. The specific CXCR4 ligand, stromal cell-derived factor-1 (SDF-1), is expressed by several tissues and upregulated by injury or ischemia. The SDF-1/CXCR4 axis plays an important role in the recruitment of circulating progenitor cells, in order to home into the sites of ischemic injury, in order to facilitate repair [27]. In most of the studies, cells have been transplanted into the subretinal space and into the vitreous cavity [28, 29]. MSCs can target and be incorporated into the neuroretinal layer. The transplanted stem cells migrated only in the injured retinal tissue, not in the normal retina [30, 31].

*(3) Biomaterials and Cell Delivery Scaffolds for the Improvement of Local Microenvironment*. In regenerative models, cells injected as a suspension often do not survive and do not reach a fully differentiated phenotype.

*(a) Extracellular Matrix Signals*. In the case of retinal reconstruction, the viability of cells delivered to the subretinal space depends on the integrity of the underlying substrate, Bruch's membrane. Safe and efficient tissue delivery must sustain survival and integration of the transplanted cells within the host and maintain the state of cell differentiation [20]. There are several studies that demonstrated the advantages of using biodegradable scaffolds, such as poly (L-lactic acid) (PLLA) and poly (D, L-lactic-co-glycolic acid) (PLGA) that could improve survival and promote differentiation of the retinal photoreceptor cells (RPCs) [32]. It is known that various factors, such as surface chemistry, mechanical properties, and surface topology, influence the attachment process and the survival of the cells. Poly(methyl methacrylate) (PMMA), poly(glycerol sebacate) (PGS), and poly(*ε*-caprolactone) (PCL) were used to produce scaffolds for retinal progenitor cell grafting. These biomaterials supported growth of murine retinal progenitor cells, both* in vitro* and* in vivo*, in degenerative mouse models [33, 34]. Recent advances in chemical synthesis and biomolecular functionalization of gold nanoparticles have led to a dramatic expansion of their potential applications in biomedicine, including biosensors, bioimaging, photothermal therapy, and targeted drug delivery [34].

*(b) Growth Factors in Retinal Regeneration*. Some growth factors have been studied extensively in retinal functional reconstruction. BDNF, ciliary neurotrophic factor (CNTF), bFGF, and neurotrophin-4 (NT-4) can prevent retinal photoreceptor cells (RPCs) from degeneration and improve RPCs recovery after being damaged. The photoreceptors' survival increased after having administered a combination of growth factors (CNTF + FGF2 or CNTF + GDNF) in an organotypic retina model [35].

bFGF is a heparin-binding protein usually tightly associated with the extracellular matrix and known to be present in the retina [36]. The relationship between bFGF and VEGFR2 (Vascular Endothelial Growth Factor Receptor 2) was proved by several studies, suggesting the implication of bFGF in the formation of retinal vessels too [37].

Another growth factor receptor, IGF-1R (Insulin Growth Factor Receptor 1), is predominantly localized in the plasma membranes of rods' outer segments. Light stress induced the activation of PI3K (phosphatidylinositide 3-kinases), through the activation and binding of IGF-1R, which led to the activation of the Akt survival pathway in photoreceptors [38]. Some transcription profiling studies identified genes that are differentially expressed in early and late retinal stem cells/progenitors. The proliferative response of the cells is correlated with the differential expression of FGF receptor 1 (FGFR1) and EGF receptor (EGFR). The proliferative maintenance of retinal progenitors includes other signaling pathways, such as those mediated by insulin-like growth factors (IGFs) and stem cell factor (SCF) [39].

## 5. Nanotechnologies as Tools in Regenerative Medicine of Retina 

### 5.1. Background

In recent years, a new field of biotechnology,* nanomedicine*, makes its way in virtually all aspects of medicine, providing new tools and possibilities, from earlier diagnosis and improved imaging to better and more efficient targeted therapies. Colloidal nanoparticles (NPs) represent one of the most promising nanotechnology products for therapeutic applications, due to their size, stability, and biocompatibility. The nanometric size of these materials allows them to be easily integrated into the biological systems. Moreover, the surface chemistry of NPs permits the conjugation with specific biomolecules which provide stability and biocompatibility, making them receptive to specific target organs and pathologic sites.

### 5.2. Arguments That Support the Use of Nanotechnology in Retinal Regeneration

The eye is a relatively isolated organ, with numerous avascular structures. The blood-retinal barrier (BRB), which is similar in structure and function with the blood-brain barrier, is selective and has two components: the inner barrier (composed by the endothelial cells, pericytes, and astrocytes) and the outer barrier (represented by the tight junctions between the RPE cells) [42]. The restrictions imposed by the BRB makes it difficult for specific drugs to reach the retina in effective concentrations. Nanoparticles (NPs) are small sized particles, less than 100 nm for at least one dimension, with various shapes (nanospheres, dendrimers, nanotubes), chemically stable, that allow the functionalization of their surface with molecules of interest [43]. In ophthalmology, nanoparticles can bring benefits by their ability to overcome the ocular barriers, as drug-delivery systems, as well as by targeting and functioning as agents for the controlled release of drugs [44]. Therefore, retinal diseases might be the main target for a nanoparticle-based therapeutic approach [45].

Yttrium oxide nanoparticles (Y_2_O_3_) administrated alone prevented photoreceptor death, by acting as free radical scavengers. They could be an alternative in the treatment of oxidative stress associated with retinal degeneration [46, 47].

Several studies aimed to use NPs for gene delivery, in the treatment of* hereditary retinal diseases. *In a murine model of retinitis pigmentosa, targeting the photoreceptor cells with CK30PEG10k-compacted DNA nanoparticles drove transgene expression in the retinal pigmented epithelium [48]. In the knock-out mice, the use of a liposome-protamine-DNA complex, as delivery for RPE 65 gene, proved its efficacy and helped the expression of RPE 65 gene for a long time [49].

### 5.3. Advantages Offered by Nanotechnology in Retinal Regeneration

The use of nanoparticles would minimize the nonspecific toxicity of drugs and enhance their therapeutic efficiency. Local administration, such as intravitreal, subretinal, or transscleral injections, is invasive and carries the risk of complications: retinal detachment, intraocular haemorrhage, intraocular infection, and cataract. Nanoparticle drug-delivery systems decrease the frequency of injections, improve efficiency, and lead to reduced side effects, resulting in improved patient compliance. Delivering therapeutic agents with nanoparticles as carriers has the following advantages: (1) the drugs can be delivered to specific cells or tissue; (2) the delivery of water-insoluble and large biomolecule drugs is improved; (3) the blood retention time is prolonged, by enhancing the drug concentration at the pathological sites and by providing sustained drug release; (4) the drug resistance mechanisms are overcome; (5) the side effects associated with conventionally used pharmaceutical excipients are reduced; (6) toxicity to healthy tissues is lowered; (7) the high surface to volume ratio of nanoparticles allows to load more drug molecules or several types of drugs simultaneously, on a single particle, as compared to the conventional carriers.

Therefore, important effort is currently invested in designing robust nanocarriers for drug delivery across the BRB, which could be safely applied* in vivo*.

### 5.4. Properties of Various Nanoparticle-Based Agents and Their Therapeutic Impact

Nanotechnology offers multiple choices for the treatment of retinal diseases. There are at least four important parameters that should be considered when designing nanoparticle-based therapeutic agents for retinal diseases: size, surface charge, shape, and the intrinsic antiangiogenic effects.

Kim et al. have reported that intravenously administered gold nanoparticles passed through the BRB, depending on their* size* [50]. They identified the small nanoparticles (20 nm) inside the retinal neurons, the endothelial cells, and the periendothelial glial cells, whereas the large ones (100 nm) could not cross the BRB.

De Jong et al. have reported that tissue distribution of gold nanoparticles is size-dependent, with the smallest 10–15 nm nanoparticles, showing the most widespread organ distribution [51].

Drug or gene release is supported for longer periods by using NPs with larger size. On the other hand, smaller size NPs are better uptaken into the cells than the larger ones, especially by endocytosis.

The affinity and internalization of NPs depend also on their* hydrophilic/hydrophobic* properties. Enhancing the uptake of NPs is possible through their functionalization with peptide ligands that interact with cells' surface receptors (such as transferrin receptor, neonatal Fc receptor) [52].* Surface charge* also influences the cellular uptake and biodistribution of nanoparticles. Generally, the positively charged nanoparticles are known to be more easily internalized than the neutral and negatively charged ones [53]. For example, Yue et al. have reported that some of the positively charged NPs escaped from lysosomes after internalization and exhibited perinuclear localization, whereas the negatively and neutrally charged NPs preferred to localize within the lysosomes [54]. The results reported by Kim et al. indicate that positive gold NPs may be more effective for drug delivery, because they are taken up to a greater extent by proliferating cells. Negative gold NPs diffuse more quickly and therefore may perform better when delivering drugs deep into the tissues is needed [55]. However, these findings are not consistent from one study to another. For example, Koo et al. have reported that cationic NPs easily penetrated the vitreal barrier and reached the inner limiting membrane, but they did not penetrate through the physical pores of the inner limiting membrane into the retinal structure. In contrast, the anionic NPs showed superior penetrating ability across the whole retina, up to the retinal pigmented epithelium [56].

The* shape* of the nanoparticles has a significant impact on their therapeutic effect, when intravenously injected. It influences the ligand targeting, cellular uptake, transport, and degradation [53, 57]. For example, Doshi et al. have reported that particles with different geometries exhibited remarkably different adhesion profiles and thereby proved the hypothesis that particle shape plays an important part in the attachment to the target site [58].

The intrinsic* antiangiogenic* properties of the inorganic nanoparticles, such as gold, silver, and silica, display synergic relationships with the drug they carry, enhancing its therapeutic effect in certain retinal diseases. There are many examples in the recent literature where inorganic nanoparticles have been used, either as therapeutic agents with antiangiogenic effects or as reliable delivery systems for targeting drugs at a specific site. For example, Kim et al. have reported that gold nanoparticles exhibit antiangiogenic effects on the retinal neovascularization involved in various vasoproliferative disorders, including retinopathy of prematurity, diabetic retinopathy, and age-related macular degeneration [59]. Jo et al. have shown that silicate nanoparticles could be considered in the treatment of retinal neovascularization, due to their intrinsic antiangiogenic characteristics [60]. Recently, Jo et al. reported that the antiangiogenic effect of gold and silica nanospheres was determined by their size, proving that 20 nm size gold and silica nanospheres suppressed* in vitro* and* in vivo* pathological angiogenesis more efficiently than their 100 nm size counterparts [61].

### 5.5. Biocompatibility of Colloidal Nanoparticles

Successful applications of colloidal nanoparticles as therapeutic agents demand their biocompatibility for the healthy tissue. In this respect, an active field of research focuses on providing a fundamental understanding of the interaction between nanoparticles and biological systems and the mechanisms of nanoparticle cytotoxicity.

Several studies suggest that the kinetic properties of nanoparticles play a crucial role on their toxicological effects. Therefore, the amount of therapeutic nanoparticles absorbed by the body and the distribution inside various organs and tissues and the clearance of nanoparticles need to be quantified carefully.

The intraretinal gold nanoparticles never affected the viability of the retinal endothelial cells, astrocytes, and retinoblastoma cells and no change was observed in the expression of representative biological molecules following the intravenous administration of gold NPs [50].

Toxicological studies have shown the distribution of particles into multiple organs, including liver, spleen, heart, kidney, lung, and brain [62–64]. The organ distribution and toxicity profile of colloidal nanoparticles result from the combination of their specific properties, such as type, size, shape, and surface chemistry. Being relatively easy to prepare in various size and shape ranges, colloidal metallic nanoparticles, particularly gold nanoparticles, represent a distinct class of nanoobjects with enormous potential for therapy. Currently, the literature contains conflicting data regarding the toxicological effects of colloidal gold nanoparticles. For example, Mochalova et al. have reported that the orally administered chitosan-coated gold nanoparticles are in themselves biologically active and exhibit adaptogenic and antioxidant properties A significant feature of this nanosystem is that the nanoparticles are completely egested from an organism within a month [63]. Simpson et al. have reported that glutathione-coated gold nanoparticles show efficient clearance through the urinary filtration system, a 100% survival rate, and no adverse histological changes, at concentrations up to and including 60 *μ*M, following subcutaneous administration. The authors suggested that glutathione may be an attractive alternative to PEG in the design of therapeutic gold nanoparticle [65]. Arvizo and coworkers have investigated the role of surface charge on pharmacokinetics, tumor uptake, and biodistribution of gold nanoparticles. They found that neutral and zwitterionic particles provide high systemic exposure and low clearance when injected intravenously and are rapidly absorbed in the systemic circulation, following intraperitoneal administration. Negative particles provide moderate systemic exposure, while positive ones are rapidly cleared [66]. The results reported by Cho et al. indicate that the 13 nm sized PEG-coated gold nanoparticles were seen to induce acute inflammation and apoptosis in the liver [67].

Based on these conflicting results, further kinetic and toxicokinetic studies are imperatively required to extend the existing knowledge on particle behavior* in vivo.*

Taken together, colloidal nanoparticles represent valuable therapeutic options for retinal diseases as they are capable to overcome BRB to reach the retina. The main characteristics of a drug nanocarrier for retinal diseases have been compiled so far: (1) small size, (2) biocompatibility, (3) monodispersity, (4) highly site specificity, and (5) consistent drug loading. In order to design an optimal therapeutic agent for retinal diseases, there are a number of crucial issues that need to be addressed, such as the effect of the nanoparticles shape, type, and surface chemistry and their interdependence.

## 6. Nanotechnology as Tool for the Improvement of Stem Cells' Transplantation in Degenerative Retinal Diseases

Nanotechnology is able to direct the differentiation of stem cells, by delivering growth factors, as bioactive molecules, that trigger specific signaling pathways [62, 68]. Retina consists of many structures containing highly specialized cells, such as sensory cells or specific neurons, bipolar cells, retinal ganglion cells, and amacrine cells. Currently, biotechnology cannot provide all the solutions to obtain this complex tissue. Understanding the pathophysiology of the retinal diseases is essential for the repair processes. Stem cells represent a powerful tool alone, but they need additional assistance by creating a proper microenvironment. During the first step, this must support the viability and proliferation of stem cells, with growth factors and extracellular matrix-mimetic materials. In the second step, stem cells must be driven to differentiate into retina-specific cells. Stem cells' niche is a micro- and nanoscale environment represented by various types of cells and chemical and physical factors. The interaction of stem cells with this niche determines their fate. This niche can be repaired or recreated with the help of NPs functionalized with proper biomolecules and micronanotopography, represented by scaffolds with mechanical properties and surface topology that resemble Bruch's membrane [63, 69].

The precise niche for retinal cells is probably very complex. For instance, neurogenesis in adult life takes place in the subventricular zone and the subgranular zone of the brain. Resident astrocytes and glial cells (considered for a long time just support cells in the brain) and their area of residence represent the neural stem cell niche [64, 70]. These requirements for recreating retinal cells' niche are closely related to the understanding of stem cells' functionality and complex interaction with the microenvironment in retinal restoration, which takes us closer to the major desideratum of retinal prosthesis.

**Figure 1:**
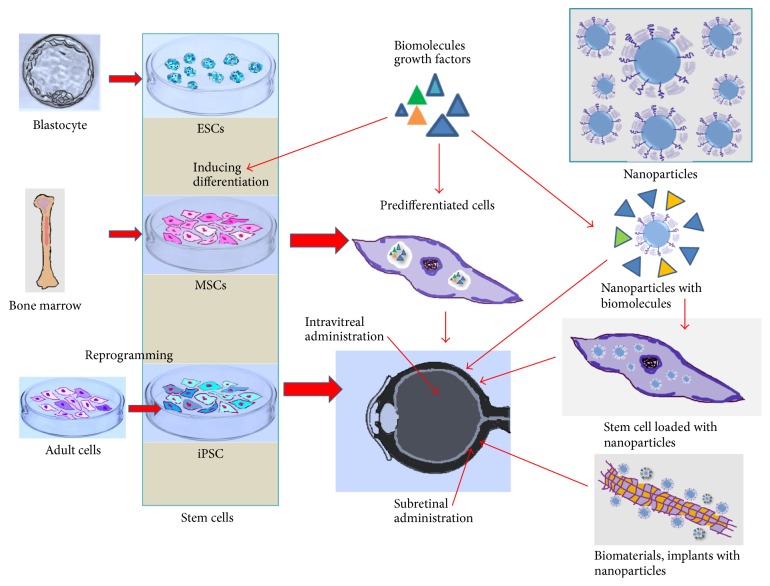
Schematic representation of stem cell transplantation strategies in degenerative retinal diseases. Embryonic stem cells (ESC), mesenchymal stem cells (MSC), and induced pluripotent stem cells (iPSCs) may be administered as undifferentiated cells (intravitreal or subretinal administration). Growth factors or other regulatory biomolecules are designed to determine the differentiation of stem cells into retinal progenitor cells or may be administered directly into the eye, to induce differentiation of resident stem cells. Another possible strategy consists of predifferentiation of stem cells under the influence of growth factors, followed by their intraocular transplantation. The conjugated biomolecules (nanoparticles and growth factors) may be administered directly into the eye but also incorporated by the stem cells, a strategy that could accelerate the differentiation process. Biomaterials, implants, and cell delivery scaffolds functionalized with nanoparticles offer the potential to develop biomedical devices, such as biosensors, bioimaging applications, photothermal therapy, and targeted drug delivery.

All the factors involved in retinal restoration, with stem cells as a central part of the processes, are summarized in Figure 1.

## 7. Difficulties and Failures

It is not clear how the differentiation process could be directed to generate specific subtypes of retinal cells. The cells' growth can be inhibited by intrinsic and extrinsic factors. A first step is to use different factors to induce cells' development to an appropriate proliferation state, both* in vitro* and* in vivo*. In the second stage, by delivering the exogenous factors, the cells are driven to differentiate into retina-specific cells. Thereafter, it seems highly difficult to generate retina-specific neurons, such as bipolar cells, retinal ganglion cells, and amacrine cells. Therefore, the creation of a permissive microenvironment is required [6].

Even if the preclinical studies proved the feasibility of using ESCs and iPSCs for treating degenerative retinal diseases associated with abnormalities in the RPE and/or photoreceptors, there are some considerations that limit their use in the clinical practice: the immunogenicity of the cells, the stability of the cell phenotype, the predilection to form tumors* in situ*, the abnormality of the microenvironment, and the association of synaptic rewiring [3].

## 8. Future Directions

 Future directions are based on the following steps:

1. the isolation and identification of stem/progenitor cells types with the highest potential to differentiate into retina-specific cells,
2. creating the permissive environment for the differentiation and integration of the stem/progenitor cells in the host retina,
3. restoration of the retinal neuronal circuits, which is necessary for the functional recovery of the retina after the transplantation procedure,
4. validation by long-term animal models for the treatment of retinal injury before translation into clinical trials.

## Figures and Tables

**Table 1:** Embryonic versus adult versus induced pluripotent stem cells for cell-based therapy.

Cell type	Advantages	Disadvantages
Embryonic stem cell	(i) Pluripotent (can form all lineages of the body: ectoderm, mesoderm, endoderm) (ii) Grown relatively easily	(i) Likely to be rejected (if donor is allogeneic, unmatched) (ii) Harbors disease-causing genes of donor Ethical problems (iii) Chromosomal errors (aneuploidy), mitochondrion DNA defects, karyotype instability [40], and risk of teratoma formation following transplantation [10]

Adult stem cell	(i) Multipotent (can form multiple cell types of 1 lineage, e.g., retinal progenitor cell) (ii) Not rejected if transplanted into donor	(i) Relatively hard to harvest (ii) Harbors disease-causing genes of donor

Induced pluripotent stem cell	(i) Pluripotent (ii) Grown relatively easily (iii) Probably not rejected if transplanted into donor	(i) May retain epigenetic features of cell type of origin (ii) Harbors disease-causing genes of donor (iii) Oncogenic potential of cells as a consequence of genetic manipulation [41]
